# Volunteering Experience of the Poor Patient Fund during Medical School

**DOI:** 10.31729/jnma.8269

**Published:** 2023-09-30

**Authors:** Saloni Shrestha, Ashima Gautam, Roshna Timilsina

**Affiliations:** 1 Nepal Medical College and Teaching Hospital, Jorpati, Kathmandu, Nepal

**Keywords:** *healthcare*, *medical students*, *volunteerism*

## Abstract

Access to healthcare poses significant financial hurdles for economically disadvantaged patients especially in developing countries like Nepal, presenting as one of the most prominent obstacles in ensuring their right to proper medical support. In response to this issue, a student-run organization called, 'Our Attempt Towards Health' has established the poor patient fund to assist those in need. The poor patient fund helps economically deprived patients with their treatment costs by providing them financial assistance and at the same time medical students have the opportunity to volunteer, creating an early and empathetic connection with patients. This experience fosters a sense of purpose and responsibility towards patients, while also honing valuable communication, counseling, and teamwork skills.

## INTRODUCTION

Poor patient fund is an initiative to help economically deprived patients by financially assisting them with treatment costs.^1^ Although financially challenged people in developing countries are reported to have high rates of illness, especially infectious diseases, there is a lack of appropriate medical care.^2^ Despite the efforts of the government of Nepal to expand free healthcare facilities to poor patients, financing remains one of the biggest challenges. Hence, nongovernmental organizations play a vital role to supplement healthcare and fill the gap.^3,4^ Our Attempt Towards Health (OATH) is a student-led organisation which runs the poor patient fund to financially assist economically deprived patients.

## EXPERIENCE

As a first-year student in a completely new environment, navigating through various student-led organizations was not an easy task. However, with its poor patient fund and its strong motive for the betterment of health, OATH, as an organization, was a preferred choice among various medical students. Engaging in volunteer work and contributing to underprivileged patients right from the first year of medical school itself gave us a profound experience of medical practice at an early stage.

Financially deprived patients who have difficulties in affording healthcare facilities like medical tests and medicines would be referred by medical professionals. After taking a brief history including sociodemographics and accompanying patient party in cases of severely illness by volunteers, OATH would financially assist the poor patient by paying for the necessary medical tests, providing the prescribed medications, and helping in discharging fees. Essential patient information, diagnosis, and financial records are documented. The poor patient fund is assisted by generous donations and various charity events. Besides charity events, the organization also holds health-related events like the annual hepatitis B vaccination program, health camps in rural areas, and health awareness programs which not only offer medical services but also provide essential health-related information to the community.

Working on a poor patient fund helped us to enhance our communication skills, counseling skills, and teamwork. As medical students, especially in the first 2 years of studying basic sciences where we do not get much patient exposure, we had the golden opportunity to come across and learn about various diseases, investigations to be sent, and treatment plans which further helps academics. The brief assessment and information gathered during the visit related to the poor patient fund enhanced our medical history-taking skills during medical school and subjective objective assessment of patient notes writing during the internship. We didn't realize it at the time back then, however, we noticed the improvement and fluency while communicating, especially while asking indirect questions about medical history during practice. These ability to compassionately interact with patients helped us in better information exchange. We were also determined on patient safety and implying prescription of cost-effective medications and investigations with help of our professors.

Having dealt with hundreds of financially deprived patients during our visit, we realized the importance of having a poor patient fund in a healthcare center and the need for healthcare equity. We believe that even a small act of kindness can make a meaningful difference in the lives of those in need. By being compassionate and reaching out to the patients, we did not only receive an opportunity to create a positive impact on their lives but also we enriched our own experiences during medical school.

## WAY FORWARD

We, as medical students, should not only be inclined towards enhancing our theoretical knowledge, however, practical skills like effective communication and empathizing with the patients should also be focused as these skills are very important components of a good medical practice. When we volunteer on initiatives like the poor patient fund, it is not just about an act of serving, in return, we learn to be more compassionate to the patients which helps us to further connect with them. Be it brainstorming ideas for charity events or managing the organization as a whole, none would have been possible without collective efforts, determination, good leadership, and teamwork. This very ability to connect with people motivates us to learn from life experiences of different people and apply to our own life for betterment. Furthermore, working on the poor patient fund from an early stage of medical school creates a focus at building interpersonal relationship with our collegues, juniors, seniors and enable us to learn more of working together in a group. Together, these practices encourage a spirit of selflessness and solidarity within the medical community.
